# Prevalence and Determinants of Long COVID Among Patients Attending the Outpatient Department of a Subdistrict Hospital in Haryana

**DOI:** 10.7759/cureus.46007

**Published:** 2023-09-26

**Authors:** Harshal R Salve, Roy A Daniel, Alok Kumar, Rakesh Kumar, Puneet Misra

**Affiliations:** 1 Centre for Community Medicine, All India Institute of Medical Sciences, New Delhi, IND

**Keywords:** india, post-covid-19, complications’, long-covid-19, post covid sequalae, covid-19

## Abstract

Introduction: Growing evidence indicates that individuals recovering from COVID-19 may experience prolonged health consequences, resulting in notable morbidity even after the acute phase. Limited published literature exists concerning sequelae of COVID-19 among the Indian population. Therefore, we conducted this study at a subdistrict hospital (secondary level) in Haryana, aiming to estimate the prevalence of long COVID and its determinants.

Methods: This hospital-based study focused on outpatients who had a confirmed history of COVID-19, with a minimum of 28 days elapsed since the positive COVID-19 diagnostic test date. We administered a semi-structured questionnaire to gather sociodemographic information, a standardized symptom assessment checklist to identify long COVID symptoms, and the Patient Health Questionnaire (PHQ-9) to evaluate and grade depression severity. Additionally, we conducted pulmonary function tests, chest X-rays, complete blood counts, and kidney and liver function tests to assess the determinants of long COVID. STATA version 14 software (StataCorp. 2015. Stata Statistical Software: Release 14. College Station, TX: StataCorp LP) was used for data analysis, and the bivariate and multivariate analyses (p-value <0.2 in bivariate analysis) were conducted to determine factors associated with long COVID.

Results: A total of 212 participants (male 53%) were recruited in this study. Among the long COVID symptoms, fatigue, body pain, cough, joint pain, and breathlessness were the most frequently reported symptoms among the study participants. The prevalence of long COVID was found to be 37.3% (95%CI: 30.7-43.8%). In the multivariate model, depression (PHQ-9 scores) AOR-1.21 (95%CI:1.07-1.35) and severity of COVID-19 adjusted odds ratio (AOR)-2.22 (95%CI:1.05-4.69) came out to be statistically significant with long COVID.

Conclusion: Findings show alarming rates of long COVID symptoms persisting in nearly 37% of COVID-19-recovered individuals. Establishing tailored guidelines is crucial to mitigate burdens and complications and enhance the quality of life for those affected.

## Introduction

The COVID-19 pandemic began in 2019 and is now a global public health problem, with 762 million cases and 6.8 million deaths recorded as of 5 April 2023 [1]. India too has been severely affected by the pandemic, with 44 million confirmed cases and 0.5 million deaths due to COVID-19 [2]. Much work has been done on the epidemiology, clinical features, and management of acute COVID-19 illness. With the passage of time, there is now emerging evidence of long-term sequelae of COVID-19, which could lead to significant morbidity in COVID-19 patients even after the resolution of the acute disease. Consensus on terminology for these long-term effects is yet to be developed. A rapid guideline from the National Institute for Health and Care Excellence (NICE) in the United Kingdom defines ongoing symptomatic COVID-19 as "signs and symptoms of COVID-19 from 4 to 12 weeks" and post-COVID-19 syndrome as "signs and symptoms that develop during or after an infection consistent with COVID-19, continue for more than 12 weeks, and are not explained by an alternative diagnosis." "Long COVID" is a broader term that includes both ongoing symptomatic COVID-19 and post-COVID-19 syndrome [3].

To date, there is a dearth of data regarding the epidemiology of post-COVID-19 sequelae in the north Indian population. As the number of COVID-19 patients recovering in India continues to rise, it becomes increasingly important to comprehend the prevalence of long COVID and its underlying determinants. Additionally, understanding the relationship between the severity of the acute disease, identifiable symptom clusters, and the development of long COVID is of great significance. The objective of this study was to investigate the distribution and determinants of long COVID among individuals who have recovered from COVID-19 and are seeking care at a subdistrict hospital in Haryana, North India.

## Materials and methods

Study setting and sample size

This cross-sectional study was conducted at a sub-district hospital in Ballabgarh block, Faridabad district, Haryana. The hospital, situated in a peri-urban area which is 35 kilometers away from the national capital, Delhi, is a 50-bed secondary-level healthcare facility. It primarily caters to the middle- and low-income population and serves as the first referral hospital for nearby primary health centers [4]. Given the exploratory nature of this study, we aimed to obtain a maximum sample size. Hence, we considered a 50% prevalence for the maximum sample size calculation, using a 95% confidence level and a relative error of 15%; the initial sample size was determined to be 178. Accounting for an additional 10% non-response rate and rounding off, the final sample size was determined to be 200.

Inclusion and exclusion criteria

All the patients attending the medicine specialty OPD (around 300 patients per day) were screened for past history of COVID-19 between March 2021 and May 2022. Those who reported COVID-19 infection in the past were assessed for eligibility and were confirmed with a positive test report to either reverse transcriptase-polymerase chain reaction (RT-PCR) or rapid antigen test (RAT). In order to understand the temporal context, only those participants with a minimum gap of at least 28 days from the date of the COVID-19-positive report were included in the study. We excluded those patients who had acute COVID-19, i.e., signs and symptoms of COVID-19 for up to 28 days. Furthermore, patients were subjected to a COVID-19 RAT test to exclude any active infection.

A pretested semi-structured interview schedule was used to collect details on socio-demography, past medical history (self-reported co-morbidity), COVID-19 testing and vaccination, and a symptoms-based checklist to diagnose long COVID, i.e., fever, cough, fatigue, loss of taste, loss of smell, nasal congestion, red eye, sore throat, headache, muscle pain, joint pain, rash, nausea/vomiting, diarrhea, shortness of breath, loss of appetite, altered mental status/reduced consciousness, chest pain/discomfort, anxiety, low mood, disturbed sleep. The interview schedule was pretested among patients attending surgery specialty OPD.

In the present study, we employed the nine-item Patient Health Questionnaire (PHQ-9) [5] to accurately evaluate and classify the severity of depression among the participants. Additionally, a comprehensive assessment was conducted, including clinical examination, anthropometric measurements, and blood sampling for both hematological analyses using the Sysmex XS-1000i (complete blood count; Sysmex Europe SE, Kobe, Japan) and biochemical investigations employing the UNICORN 480 (glycated hemoglobin, liver, and kidney function tests; Vector Biotek, Navsari, India). Throughout the laboratory analysis, stringent internal quality control procedures were implemented, and the Levey-Jennings control chart was diligently reviewed on a daily basis. Furthermore, a digital chest X-ray was performed for all enrolled individuals using the Fujifilm digital radiography, AcSelerate system (Fujifilm Holdings Corporation, Tokyo, Japan, serial number 6068).

We also conducted a pulmonary function test to assess lung capacity through a hand-held portable spirometer, MIR Spirolab A23-0J (Medical International Research, Rome, Italy), according to the standard guidelines [6]. Two measurements were performed before and at least 20 minutes after four puffs (100 mcg each puff) of salbutamol administered via metered dose inhaler with a double valve volumatic spacer with the patient in a sitting position. A separate mouthpiece was used for each participant. The data collectors received one day of training on the administration of the semi-structured questionnaire by investigators and co-investigators and another 15 days of training on the use of spirometer from the pulmonology department. A log book was maintained for all the instruments used for the data collection, and it was routinely inspected by the investigators and the co-investigators during the data collection period. The operational definitions used in this study were adapted from the NICE guidelines, and long COVID was diagnosed when all of the following criteria were met in a patient as shown in Table 1.

**Table 1:** Operational definition of long COVID

S. No.	Criteria
1.	Documentation of positive nucleic acid or antigen test for SARS-CoV-2 (28 days prior to assessment)
2.	Presence of one or more symptoms listed in the symptom checklist given below
3.	At least one symptom with a duration of four weeks or more
4.	Absence of any other cause that explains the symptoms, as judged by the examining clinician

The study protocol was approved by the Ethics Committee of All India Institute of Medical Sciences, New Delhi, India, dated 19-07-2021 and number IECPG-470/02.07.2021. Participants were recruited to the study after obtaining informed written consent, and those diagnosed with long COVID were referred to in-house medicine, physical medicine and rehabilitation, and psychiatry OPD for appropriate management. All treatments were provided to the patients free of cost.

Statistical analysis

Data was entered into Microsoft Excel (Microsoft Corporation, Washington, United States), and analysis was conducted using STATA version 14 software (StataCorp. 2015. Stata Statistical Software: Release 14. College Station, TX: StataCorp LP). The normality of the data was checked statistically by the Shapiro-Wilk test. Continuous data was represented by mean (SD) or median (IQR) based on the normality of the data, and the categorical variables were represented by frequency and percentages. Logistic regression was conducted to find the determinants of long COVID. Unadjusted and adjusted odds ratios were reported with their 95%CI. Variables with p-values less than 0.2 in bivariate analysis were included in multivariate model logistic after excluding collinear variables. A p-value of <0.05 was considered statistically significant.

## Results

**Figure 1:**
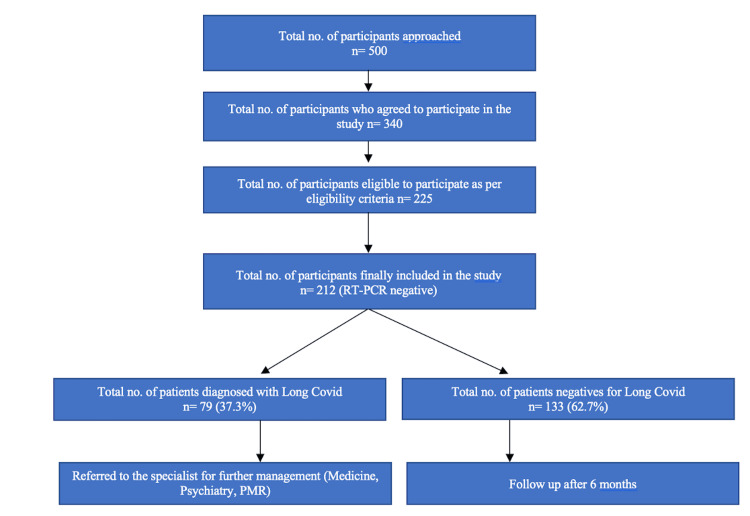
Flow of study participant selection RT-PCR: reverse transcriptase-polymerase chain reaction, PMR: physical medicine and rehabilitation

Out of the 500 individuals approached, 340 participants agreed to participate in the study, and 225 were eligible based on the inclusion and exclusion criteria. Of which 212 (53% males) were included in the study as shown in Figure 1. The mean (SD) age of the participants was 43.2 (15.2) years and 54.7% were above the age of 40 years. The mean (SD) years of education was 11.3 (3.6) with 55.2% of participants having education of more than 10 years. The median (IQR) monthly family income was Rs. 20,000 (10,000-37,500), i.e., 240 USD. Of 212 participants, 81% were currently married, 14.7% were never married, and 4.3% were widowed. Forty-six percent of the study participants were unemployed.

Based on the past medical history, it was found that hypertension, heart disease, diabetes, tuberculosis, and thyroid disorder were the most common self-reported co-morbidities among the study participants. Fatigue, fever and cough, loss of taste and smell, sore throat, and headache were the most common symptoms among the study participants. Out of 212 participants, 200 (95%) participants had received COVID-19 vaccination. Among those who were vaccinated, 193 (96.1%) participants had received a single dose and 183 (91%) participants had received two or more doses of the COVID-19 vaccine. About 66.7% of participants received the Covishield vaccine and 33.3% received Covaxin, and 97.5% of participants received vaccination from a government health facility, and only 2.5% of them received it from a private facility.

**Figure 2:**
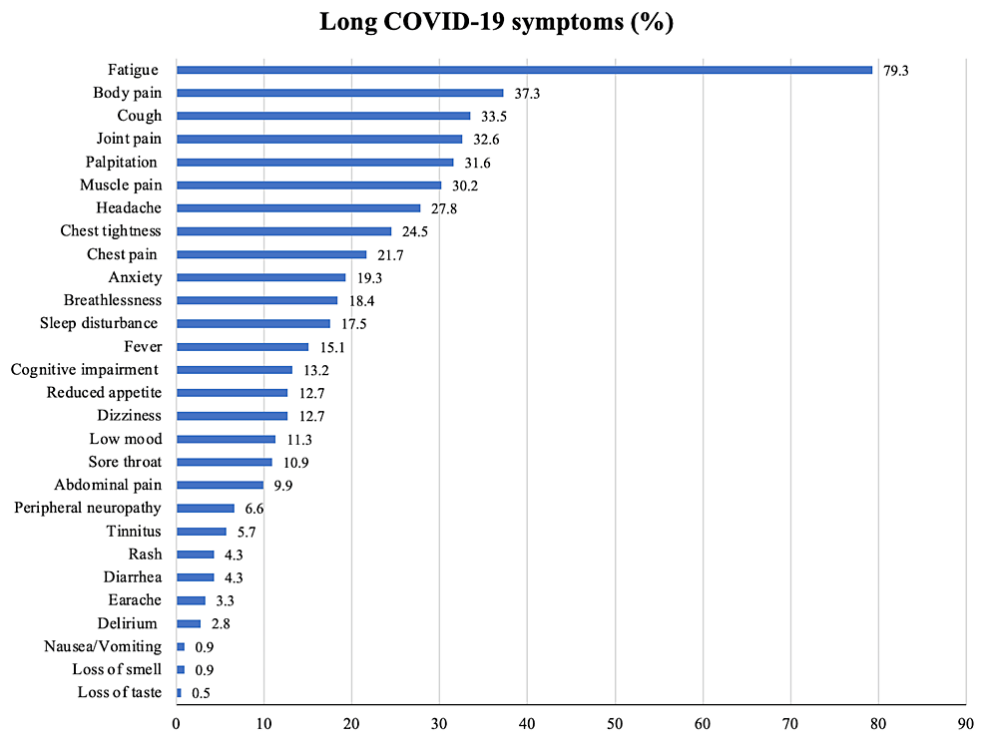
Bar chart of the distribution of participants based on long COVID symptoms

The prevalence of long COVID among all participants was determined by evaluating them using the long COVID checklist. A symptom duration of more than 28 days was considered indicative of long COVID. The analysis revealed that fatigue, body pain, cough, joint pain, and breathlessness were the most frequently reported symptoms among the study participants. The system involvement is as follows: musculoskeletal, respiratory cardiovascular, central nervous system, and gastrointestinal system as shown in Figure 2. The prevalence of long COVID in the study was found to be 37.3% (95%CI:30.7-43.8%). The prevalence among females and males was 41.4% (95%CI:31.5-51.3%) and 33.6% (95%CI:24.8-42.5%), respectively.

The median (IQR) PHQ-9 score of the study participants was 4 (2,5). Participants with long COVID exhibited higher PHQ-9 scores (5 (4,6)) compared to those without long COVID (3 (2,4)), and this difference was statistically significant (p-value <0.001). Among the study participants, eight (3.8%) of them demonstrated ECG changes, such as left ventricular hypertrophy, right atrial enlargement, and sinus tachycardia. Furthermore, abnormal chest X-ray findings were observed in 45 (21.2%) participants, including hilar lymphadenopathy, opacities in the right and left lower lungs, pleural effusion, costophrenic angle obstruction, cardiomegaly, and prominent vascular markings in both lungs. Additionally, 34 (18.4%) participants showed an FEV_1_/FVC ratio of less than 0.7. Regarding the biochemical and anthropometric parameters, no significant differences were observed between participants with long COVID and those without, except for the PHQ-9 scores, as indicated in Table 2.

**Table 2:** Distribution of participants based on biological parameters (N=212) SpO2: oxygen saturation, BMI: body mass index, PHQ-9: Patient Health Questionnaire, HbA1c: hemoglobin A1C

S. No.	Characteristics	Long COVID positive (N=79) mean (SD)	Long COVID negative (N=133) mean (SD)	Total participants (N=212)	p-value*
1.	Systolic blood pressure (mmHg)	125.6 (17.6)	128.0 (18.4)	127.1 (18.1)	0.350
2.	Diastolic blood pressure (mmHg)	81.9 (11.9)	82.3 (10.9)	82.2 (11.2)	0.811
3.	Spo2 (%)	97.9 (0.9)	97.7 (1.5)	97.8 (1.3)	0.238
4.	Pulse rate (beats/min)	87.6 (16.3)	89.2 (13.8)	88.6 (14.8)	0.448
5.	Respiratory Rate (breaths/min)	16.8 (1.6)	16.7 (1.2)	16.7 (1.4)	0.317
6.	Hemoglobin (gm/dl)	12.4 (1.9)	12.5 (1.7)	12.5 (1.8)	0.761
7.	Platelets (in lakhs)	2.1 (0.6)	2.1 (0.7)	2.1 (0.6)	0.762
8.	BMI (kg/m^2^)	25.4 (4.6)	25.3 (4.6)	25.4 (4.6)	0.895
9.	PHQ-9 score	5.3 (2.6)	3.6 (2.9)	4.2 (2.9)	<0.001
10.	HbA1c	5.8 (0.97)	5.9 (1.1)	5.9 (1.0)	0.525
11.	Serum bilirubin (mg/dl)	0.8 (0.3)	0.7 (0.2)	0.7 (0.3)	0.496
12.	Serum urea (mg/dl)	22.9 (8.5)	22.3 (8.3)	22.6 (8.4)	0.685
13.	Serum creatinine	0.7 (0.3)	0.8 (0.3)	0.8 (0.3)	0.138

In the bivariate logistic regression analysis, both the PHQ-9 score (COR-1.25, 95%CI 1.11-1.40) and the severity of COVID-19 (COR-2.80, 95%CI: 1.39-5.67) were found to be statistically significant (p-value <0.05). Subsequently, variables with a p-value of <0.2 were included in the multivariate model after adjusting for collinearity. As shown in Table 3, the same factors continued to be significant in the multivariate model, with the PHQ-9 score showing an adjusted odds ratio (AOR) of 1.21 (95%CI: 1.07-1.35) and the severity of COVID-19 showing an AOR of 2.22 (95%CI: 1.05-4.69).

**Table 3:** Bivariate and multivariate analysis of factors associated with long COVID (N=212) PHQ-9: Patient Health Questionnaire, FEV1/FCV: modified Tiffeneau-Pinelli index

Characteristics	Long COVID positive (n= 79)	Bivariate		Multivariate	
		Crude odds ratio (95%CI)	p-value	Adjusted odds ratio (95%CI)	p-value
Sex					
Male	38	Reference			
Female	41	1.40 (0.80-2.44)	0.14	1.22 (0.66-2.26)	0.51
Age					
<40 years	31	Reference			
>40 years	48	1.48 (0.84-2.60)	0.17	1.01 (0.98-1.02)	0.87
Education					
Up to 10 years	40	Reference			
>10 years	39	0.69 (0.39-1.20)	0.19	1.02 (0.52-1.98)	0.96
Marital status					
Living with spouse	67	Reference			
Living alone	11	0.59 (0.28-1.26)	0.17	0.62 (0.26-1.50)	0.288
Occupation					
Working	39	Reference			
Not working	40	1.37 (0.78-2.40)	0.27		
Body mass index (kg/m^2^)					
Underweight	5	Reference			
Normal	28	0.93 (0.29-3.10)	0.91		
Overweight/obesity	46	1.20 (0.37-3.75)	0.78		
PHQ-9 score	-	1.25 (1.11-1.40)	<0.001	1.21 (1.07-1.35)	0.002
Co-morbidity					
Absent	43	Reference			
Present	36	1.63 (0.93-2.90)	0.09	1.31 (0.69-2.48)	0.41
Number of acute COVID-19 symptoms during past COVID-19 illness					
Up to 5 symptoms	21	Reference			
More than 5 symptoms	58	1.51 (0.82-2.80)	0.19	1.11 (0.57-2.16)	0.76
Severity of COVID-19					
Non-severe	56	Reference			
Severe	23	2.80 (1.39-5.67)	0.01	2.22 (1.05-4.69)	0.04
Received 2 doses of COVID-19 vaccine					
Yes	64	Reference			
No	15	2.00 (0.90-4.39)	0.09	1.69 (0.72-3.96)	0.23
Pulmonary function test					
Normal (FEV_1_/FCV>0.7)	151	Reference			
Abnormal (FEV_1_/FCV<0.7)	34	1.74 (0.82-3.70)	0.15	1.72 (0.75-3.96)	0.20

## Discussion

This hospital-based cross-sectional study was conducted to estimate the prevalence of long COVID using a standardized symptom checklist and to determine the associated factors. The study revealed the prevalence of long COVID as 37.3% (95%CI:30.7-43.8%). The prevalence was slightly higher among females, with a prevalence of 41.4% (95%CI:31.5-51.3%). The mean age of the study participants was 43 years, which exceeded the mean age reported in other studies with similar objectives [7,8].

Our study found that hypertension, diabetes, and heart disease were prevalent co-morbidities. COVID-19 and non-communicable diseases (NCDs) interact, heightening vulnerability both ways. Future pandemics emphasize the need to understand syndemic drivers and establish safeguards. Integrating holistic care for NCD-affected individuals into national responses is crucial. Swift and urgent action is necessary, focusing on syndemic prevention. Collaborative, data-driven efforts are essential to address post-pandemic health disparities effectively [9].

The most common symptoms during the past COVID-19 illness among the study participants were fatigue (93%) followed by fever (91.5%) and cough (84.4%). This pattern is similar to the study conducted by Arjun et al. [8] Multiple studies have documented symptoms persisting for as long as 12 weeks after onset of COVID-19, in patients managed outside hospital settings. Evidence suggests that fatigue, cough, and breathlessness were the most common symptoms of long COVID infection [10]. In this study, a similar pattern was seen, and this is similar to other studies conducted in India [7,8]. The symptoms self-reported through the COVID Symptom Study app and the United Kingdom's National Coronavirus Infection Survey have documented fatigue as the predominant symptom [11]. Several systematic reviews and meta-analyses focused on long COVID have consistently identified fatigue as either the most frequent symptom or one of the top three symptoms experienced [12,13].

Impairments in multiple organ systems, including persistent abnormalities in chest imaging and lung function, have been observed in various studies conducted globally [14,15]. Emerging data also suggest that these sequelae impair and reduce the quality of life [16]. In our study, 21% of the patients had an abnormality in the chest X-ray, and 18% of the participants had an FEV_1_/FVC value of less than 0.7. This shows that there is an impairment in the lung and cardiac functions following COVID-19 infection. Hence, separate guidelines are needed to be formulated for optimal management of post-COVID-19 syndrome.

A study conducted by Sarda et al. [7] in New Delhi published in 2022 among COVID-19-positive patients through telephonic interviews found the prevalence of long COVID as 28.2% (95%CI:23.0-34.2). Another similar study conducted by Arjun et al. [8] in Bhubaneswar published in 2022 among COVID-19-positive patients estimated the prevalence of long COVID (delta variant) as 29.2% (95%CI:25.3%, 33.4%). Both these estimates are lower when compared to our study’s estimate.

However, our study's estimates revealed a higher prevalence of long COVID when compared to the findings of both Sarda et al. [7] and Arjun et al. [8]. Several factors may underlie this disparity. Firstly, our study's population featured individuals with a relatively higher mean age of 43 (15.2) years, in contrast to the mean ages of 35 and 39 years observed in the other studies. This divergence in age distribution is particularly relevant because age has been established as a pivotal factor associated with both severe COVID-19 and the risk of developing long COVID [17,18]. The presence of a more elderly demographic in our study may have contributed to the elevated long COVID prevalence observed.

Moreover, the variations in study timeframes and the prevalent variants of COVID-19 in the respective regions where these investigations were conducted should not be overlooked. These factors play a substantial role in influencing the severity of the illness and, consequently, the likelihood of developing long COVID. As the virus has mutated over time, with the emergence of variants such as the delta variant, the clinical course and outcomes of COVID-19 have been subject to fluctuations. In addition, the emerging evidence in the field of COVID-19 research has illuminated the connection between BMI and the severity of the disease [19]. Notably, our study reported a relatively high rate of overweight and obese individuals, totaling 54%. This figure exceeds the proportions observed in the investigations led by Sarda et al. [7] and Arjun et al. [8]. This disparity may also contribute to the elevated prevalence of long COVID in our study, as individuals with higher BMIs are more prone to experiencing severe COVID-19 symptoms.

In the multivariate analysis, long COVID and the severity of COVID-19 with an adjusted odds ratio of 2.22 (95%CI:1.05- 4.69) came out to be statistically significant. The study done by Arjun et al. [8] also showed a statistically significant association (AOR-5.71 (95%CI:3.00,10.89)) of long COVID with the severity of COVID-19 illness. The presence of co-morbid conditions is an important risk factor for long COVID [20]. Studies showed a significant association of long COVID with pre-existing illness [7,8]. The greater risk of long COVID with any pre-existing disease was also seen in our study; however, it was not statistically significant.

BMI, COVID-19 vaccination status, and number of symptoms during the past COVID-19 illness are important predictors of long COVID [21]. The studies by Arjun et al. [8] and Sarda et al. [7] showed significant results on these associations. A systematic review by Iqbal et al. [22] also found that hospitalization and severity were important predictors of long COVID. The reason for a non-significant result in this study might be due to the comparatively lower sample size in our study. Several studies found a significant association between long COVID and impaired lung function [23]. We assessed the association between long COVID and lung function through a pulmonary function test. Participants in our study with decreased lung function (FEV_1_/FCV 0.7) were associated with long COVID, but this association was not statistically significant.

One of the important clinical manifestations of long COVID is depression, anxiety, and cognitive dysfunction [24]. We evaluated the association between long COVID and depression using a PHQ-9 questionnaire. There was a statistically significant association between long COVID and depression, AOR-1.21 (95%CI:1.07-1.35). This is similar to another study by Goodman et al. [25] where they found a significant association with depression, anxiety, and suicide ideation. The findings from this study provide crucial insights for policymakers at the state and national levels, facilitating the development of targeted policies to effectively address the post-COVID-19 issues that arise within the population.

Strengths and limitations

The present study fills the gap of the limited literature on long COVID and its determinants among the peri-urban population in north India. In order to minimize misclassification bias, we exclusively included patients with confirmed COVID-19 using RT-PCR reports. The data collectors underwent comprehensive training, and laboratory investigations were conducted with daily internal quality control measures, thus enhancing the validity of our study. We used a standardized methodology to assess both the exposure and the outcome. Although we achieved a high response rate, it is important to exercise caution when generalizing the findings to the broader population as this study was conducted in a hospital setting. Additionally, it should be noted that this study was not specifically designed to identify the factors associated with long COVID-19. Given the cross-sectional nature of our study, we were unable to establish temporality, and the self-reported co-morbidities were collected after the COVID-19 infection and the morbidities before the COVID-19 infection were not objectively measured that might have an impact on the prevalence of long COVID. We did not assess other potential predictors of long COVID, such as mental health and quality of life.

## Conclusions

This study reveals a concerning finding that nearly 37% of individuals who have recovered from COVID-19 continue to experience long COVID symptoms, which is indeed alarming. Depression was assessed using PHQ-9 and the severity of COVID-19 was significantly associated with long COVID. To comprehensively understand the factors contributing to long COVID, further longitudinal studies are warranted. In light of the pressing need to address long COVID, it is crucial that we establish distinct and specialized guidelines tailored explicitly to this condition. These guidelines should encompass a holistic approach, encompassing medical, psychological, and social dimensions of care. By doing so, we can effectively alleviate the burdens and complications associated with long COVID, ultimately leading to a tangible improvement in the quality of life for the individuals who continue to grapple with its aftermath.
